# The role of HECT-type E3 ubiquitin ligases in inflammation

**DOI:** 10.3389/fimmu.2026.1810360

**Published:** 2026-04-15

**Authors:** Ziyi Wang, Peirui Wang, Huawang Xie, Zhibo You, Xiping Liu, Fang Cao, Shengtao Yao

**Affiliations:** 1 Department of Neurosurgery, The Affiliated Hospital of Zunyi Medical University, Zunyi, Guizhou, China; 2 Department of Biochemistry, Zunyi Medical University, Zunyi, Guizhou, China

**Keywords:** HECT-type E3 ubiquitin ligases, HERC subfamily, inflammation, NEDD4subfamily, other HECTs, signaling pathways, ubiquitination

## Abstract

Homologous to the E6-AP Carboxyl Terminus (HECT)-type E3 ubiquitin ligases are key components of the ubiquitin-proteasome system (UPS) and play an important role in the regulation of inflammatory responses. Inflammation serves as a core defense mechanism of the host against infection and tissue damage, while its dysregulated and unresolved activation drives the pathogenesis of diverse chronic inflammatory diseases. In recent years, the functions of HECT-type E3 ubiquitin ligases in inflammatory signaling pathways have gradually been revealed, particularly the differential roles of members of the HECT and RCC1-Like Domain Containing E3 Ubiquitin Protein Ligase (HERC) and Neural Precursor Cell Expressed Developmentally Downregulated 4 (NEDD4) subfamilies in immune cell activation, cytokine expression, and ubiquitination modifications. Although some molecular mechanisms of certain HECT-type E3 ubiquitin ligases have been reported, the complex regulatory networks and mechanisms of action of these ligases remain largely unelucidated, with conflicting research conclusions, unclarified cell type-specific functional heterogeneity, and notable limitations in current research systems remaining unresolved. This article systematically reviews the research progress of HECT-type E3 ubiquitin ligases in inflammation, focusing on the functional characteristics of different subfamilies and their molecular mechanisms in regulating inflammatory processes, and further conducts a critical analysis of conflicting findings in existing studies, the limitations of current research, and the context-dependent functions of specific ligases in different cell types. It aims to provide theoretical support and research directions for a deeper understanding of the biological functions of HECT-type E3 ubiquitin ligases and their application as potential therapeutic targets in inflammation-related diseases.

## 1 Introduction

The ubiquitin-proteasome system (UPS) plays a crucial role in protein degradation and cell signaling within cells, where E3 ubiquitin ligases (hereinafter referred to as E3 ligases) are the main enzymes responsible for recognizing specific substrates and catalyzing the transfer of ubiquitin, thereby regulating various biological processes such as the cell cycle, immune response, and cell signaling (1–3). HECT (Homologous to the E6-AP Carboxyl Terminus)-type E3 ubiquitin ligases employ the catalytic mechanism involving thioester intermediates and obvious substrate specificity, which makes them key regulatory factors for cellular homeostasis and disease-related pathways (4, 5).

Inflammation is the body’s defense response to infection and tissue damage through interrelated signal cascades and immune cell regulation (6). Ubiquitination is a major post-translational modification that is essential for inflammatory signal transduction and immune cell function. In the process of inflammation, E3 ubiquitin ligases affects the stability and activity of Nuclear Factor kappa-light-chain-enhancer of activated B cells (NF-κB), Mitogen-Activated Protein Kinase (MAPK), and other signal molecules. This regulation affects the different stages of inflammatory responses (7–9). Therefore, the ubiquitination network is crucial to maintaining the balance of inflammatory responses, and the abnormality of the network may lead to chronic inflammation and autoimmune diseases (10–12).

**Figure 1:**
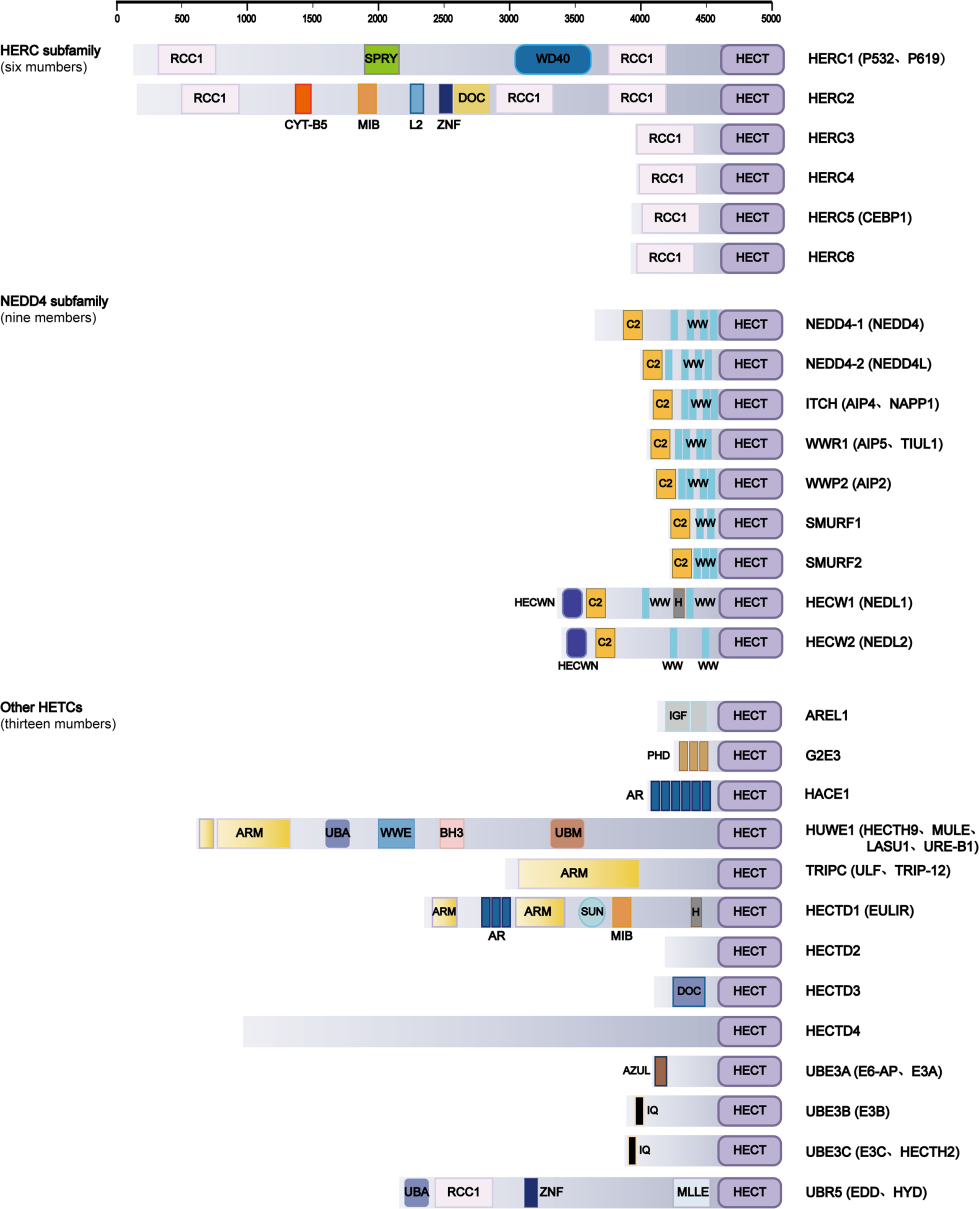
The domain architecture of HECT-type E3 ligases. The domain abbreviations used are as follows: HECT, homologous to E6AP C-terminus; NEDD4, neural precursor cell expressed developmentally downregulated 4; HERC, HECT and RLD domain containing E3 ubiquitin protein ligase; ITCH AIP4, NEDD4 family-interacting protein 1; WWP, WW domain containing E3 ubiquitin protein ligase; SMURF, SMAD ubiquitin regulatory factor; HECW, HECT, C2, and WW domain containing E3 protein ligase; AREL1, apoptosis-resistance E3 ubiquitin protein ligase 1; G2E3, G2/M phase-specific E3 ubiquitin protein ligase; HACE, HECT domain and ankyrin repeat containing E3 ubiquitin protein ligase; HUWE, HECT, UBA, and WWE domain containing E3 ubiquitin protein ligase; TRIP12(TRIPC), thyroid hormone receptor interactor 12; HECTD, HECT domain E3 ubiquitin protein ligase; E6AP(UBE3A), E6-associated protein; UBE3B, ubiquitin–protein ligase E3B; UBE3C, ubiquitin protein ligase E3C; UBR5, ubiquitin protein ligase E3 component N-recognin 5. Predicted domains are abbreviated as follows: RCC1-like domain, Regulator of chromosome condensation 1/beta-lactamase-inhibitor protein II; SPRY, B30.2/SPRY domain, concanavalin A-like lectin/glucanase domain; WD40, WD40/YVTN repeat-like-containing domain; CYT-B5, Cytochrome B5-like heme/steroid binding domain; MIB, MIB-HERC2 domain; L2, ribosomal protein L2 domain; ZNF, zinc finger; DOC, APC10/DOC domain, galactose-binding domain-like domain; C2, C2 domain; WW, WW domain; HECWN, HECW1/2 N-terminal domain; H, helical bundle (HECTD1) and helical box domain (HECW1); IGF, immunoglobulin-like fold; PHD, PHD-type zinc finger; AR, ankyrin repeat-containing domain; ARM, armadillo-type fold domain; UBA, ubiquitin-associated domain; WWE, WWE domain; BH3, BCL-2 homology region 3 domain; UBM, ubiquitin-binding motif; SUN, SAD1/UNC domain; AZUL, AZUL domain/N-terminal zinc-binding domain; IQ, IQ domain/EF-hand binding site; MLLE, Mademoiselle/PABC domain. Predicted coiled-coil regions are not included. Protein domain prediction analysis was performed using Interpro server. Approximate domain sizes and positions are scaled along the primary sequence of the protein.

The HECT-type E3 ubiquitin ligases family is usually divided into the HERC subfamily, the NEDD4 subfamily, and other HECTs (13). **Figure 1** shows all members of the HECT-type E3 ubiquitin ligases. These subfamilies show significant functional differences in inflammation. For example, in the NEDD4 subfamily, WWP2 reduces myocardial inflammation and fibrosis by promoting the ubiquitination-dependent degradation of PARP1, which highlights the role of HECT-type E3 ubiquitin ligases in limiting inflammation-related tissue remodeling (14). In addition, the HECT-related protein NEDD4 Binding Protein 1 (N4BP1) negatively regulates the activation of NF-κB in the Tumor Necrosis Factor (TNF) signaling pathway by recognizing linear ubiquitin chains. This reveals the different regulatory capabilities of HECT family members in signaling complexes (15). HERC subfamily members and other HECT-type E3 ubiquitin ligases regulate immune pathways through different mechanisms, further emphasizing the breadth of HECT-mediated inflammatory responses.

In summary, the role of HECT-type E3 ubiquitin ligases in inflammation and immunomodulation is complex and dynamic. The current research, combined with the latest progress of molecular biology and immunology, has begun to reveal the specific regulatory processes of various HECT-type E3 ubiquitin ligases from different subfamilies involved in inflammatory signal transduction and immune function (16). This progress provides a theoretical basis for understanding the mechanism of inflammation. These findings not only improve the understanding of the mechanism of inflammation, but also promote the development of treatment methods for HECT-dependent nodes in immune and inflammatory diseases (5, 16, 17). However, existing studies remain mostly descriptive, with insufficient critical analysis of conflicting results, systematic evaluation of research limitations, and in-depth interpretation of cell type-specific functional heterogeneity. The present review is dedicated to resolving two core scientific questions: the cooperative and antagonistic mechanisms of different HECT subfamilies in the regulation of inflammatory signaling, and the core determinants that define the pro-inflammatory versus anti-inflammatory functional orientation of a specific HECT-type E3 ubiquitin ligase across varied cellular contexts. Therefore, this review not only summarizes the regulatory mechanisms of various HECT-type E3 ubiquitin ligases in inflammation across different subfamilies, but also conducts a critical dissection of the above key issues, which is crucial for drawing a HECT-centered regulatory network and promoting precise immunomodulation strategies.

## 2 The role of the HERC subfamily in inflammation mechanisms

### 2.1 Structure and functional characteristics of HERC subfamily members

The HERC subfamily is an important part of HECT-type E3 ubiquitin ligases, which have a unique structure and multifunctional regulatory ability. Members of the HERC subfamily usually include a C-terminal HECT domain and multiple N-terminal RCC1-like domains (RLDs) (18). The RLD domains participate in protein recognition and substrate binding, which endow HERC subfamily members with high substrate specificity and functional diversity (19).

Specifically, HERC subfamily members can be divided into two categories according to their size and structure: large HERC (such as HERC1 and HERC2) and small HERC (such as HERC3 to HERC6) (20, 21). Due to their large size and complex structure, large HERCs contain multiple RLD domains. Consequently, they not only play a role in ubiquitination modification, but also in the process of signal regulation, DNA damage response, neurodevelopment, and immunity (20, 22, 23). For example, HERC1 and HERC2 have dual functions in the development of the nervous system and tumorigenesis. They can be used as both tumor suppressors and potential oncoproteins of certain tumor types (20). This dual function stems from their complex interactions and the adjustment mechanism of multiple domains, mainly HECT and RLD domains (22).

Functionally, the RLD domains act as a module that regulates the assembly of protein complexes, mediates the interaction with various signaling molecules to realize the selective recognition and regulation of ubiquitin substrates (20). Notably, small HERC members such as HERC5 and HERC6 participate in antiviral immune responses other than standard ubiquitination. They regulate the activation of Stimulator of Interferon Genes (STING) and NOD-like Receptor Family Pyrin Domain Containing 3 (NLRP3) inflammasomes through ISGylation (a ubiquitin-like post-translational modification) to prevent their excessive degradation and promote antiviral immunity (24–27).

In addition, members of the HERC family also play an important role in regulating cellular protein homeostasis (20, 22). Different domains can recognize multiple substrate objects at the same time (28–30). They regulate the stability and activity of these substrates through ubiquitination and Interferon-Stimulated Gene 15 conjugation (ISGylation) modification, thus participating in the fine-tuning of the intracellular signaling pathways (31). For example, HERC2 regulates the degradation of β-catenin through ubiquitination and indirectly controls the expression of the intracellular drug-metabolizing enzyme Cytochrome P450 Family 2 Subfamily E Member 1 (CYP2E1), helping to protect the liver from drug-induced damage (32). In hematological malignancies, HERC1 shows a unique regulatory effect in chronic myeloid leukemia through interaction with the Breakpoint Cluster Region-Abelson Murine Leukemia Viral Oncogene Homolog 1 (BCR-ABL1) fusion protein. The expression level of HERC1 is closely related to the state of the disease (33).

In summary, the HECT domain confers catalytic ubiquitin transfer activity (18), while multiple RLD domains provide rich proteins for interaction interfaces (22, 34, 35). These characteristics enable them to regulate various cell processes, including signal transduction, protein homeostasis, immune response, and cell proliferation (36). This diversity of structure and function makes the HERC family play a key role in maintaining cellular homeostasis and responding to inflammation, and also provides potential molecular targets for targeted treatment (37).

### 2.2 The HERC subfamily regulation of inflammatory signaling pathways

Members of the HERC subfamily mainly regulate the transmission of inflammatory signaling through ubiquitination and ubiquitin-like modification to control the stability and activity of inflammatory mediators (37–40). Large HERC ligases, including HERC1 and HERC2, can affect NF-κB signaling and other inflammatory pathways through ubiquitinating key pathway components (41, 42). The study found that HERC2 promotes the K33 ubiquitination of key regulatory proteins such as TANK Binding Kinase 1 (TBK1) through direct interaction, activates the downstream transcription factors Interferon Regulatory Factor 3 (IRF3) and IRF7, enhances the expression of type I interferon, and enhances the antiviral inflammatory responses (37, 43, 44). Consistent with this effect, the expression of HERC2 in the liver tissue of patients with chronic hepatitis B is significantly reduced, and liver-specific HERC2 knockout mice are more sensitive to hepatitis virus infection, suggesting its key regulatory role in antiviral inflammation. In addition, through ubiquitination of β-catenin, HERC2 regulates the transcription of CYP2E1, limiting drug-induced liver injury, suggesting its protective effect in inflammation-related liver injury (32).

As an interferon-induced E3 ubiquitin ligase, HERC5 mainly mediates ISGylation modification, covalently attaching Interferon-Stimulated Gene 15 (ISG15) molecules to the target protein, and regulates the antiviral inflammatory responses (32, 45). ISGylation is an important post-translational modification that stabilizes inflammatory signal molecules by preventing the degradation of the UPS (46–48). Recent studies show that HERC5-dependent ISGylation promotes the activity of NLRP3 inflammasomes by enhancing the stability of NLRP3. In terms of mechanism, Toll-like receptor (TLR) activates and induces NLRP3 ISGylation, prevents K48-linked ubiquitination-driven degradation, and maintains inflammasome function. Viral infection including Severe Acute Respiratory Syndrome Coronavirus 2 (SARS-CoV-2) and type I interferon upregulate ISG15 and HERC proteins, thus increasing NLRP3 ISGylation and possibly exacerbating inflammatory responses. Accordingly, HERC6 gene knockout mice exhibited attenuated NLRP3-dependent inflammation, indicating that HERC-mediated ISGylation is an important mechanism governing inflammatory intensity and pathogenesis (24).

In summary, members of the HERC subfamily regulate inflammatory signaling by controlling the ubiquitination and ISGylation state of key pathway proteins, thus affecting the intensity and persistence of inflammatory responses (24). Although the roles of HERC1 and HERC2 in proliferation, genomic maintenance, and other homeostasis processes are best known (33, 49), emerging data support their participation in immunomodulation by regulating ubiquitin-dependent signals (20, 50, 51). For example, the expression of HERC1 is related to the differentiation process of hematopoietic cells, and its expression level in chronic myeloid leukemia decreases, suggesting its potential regulatory role in inflammation and the tumor microenvironment of the hematopoietic system (33, 52). Notably, current studies on HERC2 have presented conflicting conclusions regarding its inflammatory regulatory effects: HERC2 enhances antiviral inflammatory responses by promoting K33 ubiquitination of TBK1 in hepatocytes, while it exerts an anti-inflammatory effect by degrading NOD-like Receptor Family CARD Domain Containing 4 (NLRC4) in microglia and NCOA4 in macrophages. This bidirectional regulation is mainly driven by cell type-specific substrate expression and distinct ubiquitin chain linkage types it catalyzes, and the lack of cell type-specific knockout models in most studies has led to an ambiguous definition of its core inflammatory function *in vivo*. Therefore, the HERC subfamily plays a key role in the regulation of inflammatory signaling pathways and inflammatory responses through various ubiquitination modifications, providing potential targets for exploring the treatment of inflammatory-related diseases, while the unresolved conflicting findings and cell type-specific functional heterogeneity also highlight key directions for future research.

### 2.3 Regulatory roles of the HERC subfamily in immune cell functions

In addition to the central role in signaling, HECT-type E3 ubiquitin ligases directly shape the immune cell program. Large (HERC1/2) and small (HERC3-6) HERC members regulate the immune response by regulating ubiquitination and ubiquitin-like modifications in immune cells (20, 53). In macrophages, HERC2 is involved in regulating macrophage inflammatory polarization and cytokine secretion. HERC2 regulates the ubiquitination pathway in macrophages, affecting the stability and activity of key signal molecules and transcription factors. By regulating the expression of pro-inflammatory cytokines such as Tumor Necrosis Factor alpha (TNF-α) and Interleukin 1 beta (IL-1β), it promotes the secretion of anti-inflammatory cytokines such as Interleukin 10 (IL-10), thus maintaining the dynamic balance of the immune response, preventing excessive inflammation and causing tissue damage (54, 55).

The role of HERC5 in dendritic cells has also received wide attention (56). Dendritic cells are an important bridge between innate immunity and adaptive immunity; their antigen presentation function determines the efficiency of starting the immune response (57–59). HERC5 can bind ISG15 to the target protein by mediating ISGylation to regulate the antigen processing and presentation process, enhancing the ability of activated dendritic cells to present antigen peptides to T cells (60–62). This regulation promotes the activation of adaptive immunity and the coordinated control of inflammatory responses. In the context of viral infection, interferon-driven HERC5 is upregulated to support antiviral defense programs and may reshape inflammation signaling at the same time (24).

In summary, HERC ligases coordinate immune cell functions through ubiquitination and ISGylation, which have an important impact on the activity and duration of inflammation of inflammasomes (20, 24). The inflammatory functions of HERC subfamily members exhibit significant cell type context dependence: HERC2 exerts an anti-inflammatory effect in macrophages and microglia by inhibiting inflammasome activation, while it plays a pro-inflammatory role in hepatocytes during antiviral infection by enhancing type I interferon signaling; HERC5/6 mainly exerts a pro-inflammatory effect in macrophages and dendritic cells by mediating NLRP3 ISGylation and regulating antigen presentation. These findings reveal the complex, multilayered regulatory network formed by members of the HERC subfamily in immune cells, indicating that they are key factors in maintaining immune homeostasis and potential treatment targets for inflammation-related diseases (20). However, current research has notable limitations: most functional studies of HERC family members are limited to macrophages and dendritic cells, while their regulatory roles in neutrophils, adaptive immune T/B cells, and tissue parenchymal cells remain largely unelucidated; in addition, most studies on HERC1/3/4 only focus on their non-inflammatory functions, and their roles in inflammatory regulation are still in the preliminary exploration stage, forming a significant research gap. An in-depth analysis of the fine-tuning mechanism of HERC subfamily members in different immune cells in the future will help formulate precise treatment strategies for inflammation and autoimmune diseases. **Table 1** summarizes the details of the HERC family, including their substrates, interacting partners, cell types, inflammatory effects, related pathways, and functions in Inflammation, with additional supplementation of cell type-specific functional characteristics and conflicting research conclusions.

**Table 1:** HERC subfamily: inflammatory characteristics and regulators.

HERC subfamily	Substrate	Cell types	Inflammatory effect	Known linkage type	Pathway	Biological function	Reference
HERC2	β-catenin	hepatocytes/HepaRG	Anti-	K48	HERC2-β-catenin-CYP2E1	DNA damage, oxidative stress, inflammatory responses	(32)
	TBK1	hepatocytes/HepaRG	Anti-	K33	HERC2-TBK1-IRF3/7		(37)
	NCOA4	THP-1/hMDM, BMDMs	Anti-		TRIM21-HERC2-NCOA4		(54)
	NLRC4	BV-2	Anti-	K48	Fendrr-HERC2-NLRC4		(55)
HERC5/HERC6	NF-κB	KYSE30, KYSE150	Pro-		circJPH1-XRCC6-NF-κB -HERC5	Innate immune, inflammatory responses	(63)
	NLRP3	PMs, THP-1	Pro-	K48	HERC5/6-ISG15-NLRP3		(24)

## 3 Mechanisms of NEDD4 subfamily action in inflammation

### 3.1 NEDD4 subfamily structure and substrate specificity

The NEDD4 subfamily forms a class of HECT-type E3 ubiquitin ligase with a conserved modular structure: N-terminal C2 domain, multiple central WW domains, and C-terminal HECT catalytic domain (13, 64). This structural feature gives the NEDD4 subfamily unique functional characteristics and substrate recognition ability (65). The C2 domain mainly mediates the binding with the cell membrane, so that the NEDD4 protein is located in a specific region and participates in the membrane-related signal transduction and protein endocytosis process (66). The WW domain achieves specific binding with different substrates by identifying the phosphorylated serine/threonine residues in the typical PY motif (PPxY or LPxY) and substrate protein (67–69), which enhances the substrate diversity and regulatory flexibility of NEDD4 subfamily members (70).

By coordinating domain function, NEDD4 ligases regulates membrane receptor signaling, protein endocytosis, and intracellular transport (71–74). For example, NEDD4 is located in the plasma membrane through the C2 domain, and the PY motif of the receptor is identified by the WW domain (66). Then it promotes receptor ubiquitination modification, internalization, and degradation, thus weakening the signal strength and forming a cellular response (75–77). Structural differences between different NEDD4 members, such as the lack of part of the C2 domain of NEDD4-1’s specific shear isomer NEDD4-1, reduce its autoinhibition effect, enhance catalytic activity, and change the substrate binding characteristics (78). Therefore, the substrate specificity of the NEDD4 subfamily is not only driven by the substrate sequence, but also related to the environment (79–81). For example, NEDD4L (NEDD4-2) regulates the malignant progression of esophageal cancer through its HECT domain-specific binding and ubiquitinating ITGB4 protein, which illustrates its function and substrate specificity in the disease (82). In myocardial cells, NEDD4L regulates the ubiquitination of the sodium channel NaV1.5, affecting the electrical signal transduction of myocardial cells (83). Collectively, NEDD4 subfamily members are involved in diverse cellular processes, including bone and tooth development, immune regulation, and tumor progression, reflecting the multifunctional properties conferred by their modular domain architecture (70, 84).

### 3.2 NEDD4 subfamily regulation of inflammatory signaling pathways

The NEDD4 subfamily includes NEDD4-1/2, Itchy E3 Ubiquitin Protein Ligase (ITCH), SMAD Specific E3 Ubiquitin Protein Ligase 1/2 (SMURF1/2), WW Domain Containing E3 Ubiquitin Protein Ligase 1/2 (WWP1/2) and HECT, C2 and WW Domain Containing E3 Ubiquitin Protein Ligase 1/2 (HECW1/2). These enzymes regulate the intensity and duration of inflammatory responses through ubiquitination, thus maintaining immune and tissue homeostasis. Research shows that the NEDD4 subfamily has multiple functions and mechanisms in the inflammatory signaling pathways (85, 86).

As a key representative of the NEDD4 subfamily, NEDD4–1 is mainly characterized as a negative regulatory factor to prevent excessive inflammation in most current studies. Research shows that NEDD4–1 inhibits excessive inflammatory responses by regulating the adaptor proteins in the TLR signaling pathway (87). In the atherosclerosis model, NEDD4–1 promotes the K48 ubiquitination of the GATA4-p62 complex by upregulating the Retinoid X Receptor alpha/Peroxisome Proliferator-Activated Receptor gamma (RXRα/PPARγ) axis, inhibiting the production of senescence-associated secretory phenotype (SASP) inflammatory factors, and reducing inflammatory responses in primary macrophages (88). In the acute lung injury model, NEDD4–1 reduces lung inflammation and oxidative damage by enhancing the ubiquitination of Notch1 in alveolar epithelial type II cells, which has a protective effect (89). These findings show that NEDD4–1 regulates the occurrence and progression of inflammation by directly acting on the ubiquitination of key proteins in innate immune cells and tissue parenchymal cells, thus preventing excessive immune response and tissue damage (90–92). In addition, in the viral and bacterial co-infection model, NEDD4–1 inhibits the activation of NLRP3 inflammasomes and regulates the process of Gasdermin D (GSDMD)-related inflammatory cell death in macrophages, and jointly inhibits excessive inflammation (93, 94). In addition, NEDD4–1 inhibits inflammation and endothelial dysfunction induced by oxidized low-density lipoprotein (ox-LDL) by regulating oxidative stress and apoptotic signaling, and protects the function of vascular endothelial cells (95). However, conflicting conclusions have been reported in existing studies: in HeLa and HEK-293T cell lines, NEDD4–1 promotes K48-linked ubiquitination and degradation of IκBα via TMEPAI, thereby activating the NF-κB signaling and exerting a pro-inflammatory effect (96). The core reasons for this functional discrepancy include: first, the difference in cell types, with anti-inflammatory effects mostly verified in primary immune cells and parenchymal cells with physiological relevance, while pro-inflammatory conclusions are mostly derived from immortalized tumor cell lines with genetic background aberrations; second, the difference in substrate preference determined by the cellular microenvironment, with NEDD4–1 targeting different substrates in distinct cell types to achieve bidirectional regulation of inflammation. Current studies have not yet clarified the dominant function of NEDD4–1 in physiological inflammatory states *in vivo*, which is a key limitation of current research.

NEDD4L also shows different inflammatory functions through several routes, with significant cell type context dependence and partial conflicting conclusions in existing studies. It can inhibit the Interleukin 17 (IL-17) receptor signaling pathway and limit the activation of p38/NF-κB by promoting the ubiquitin-dependent degradation of mitogen-activated protein kinase 2 (MEKK2) in mouse embryonic fibroblasts and astrocytes, and negatively regulating the TNF-α, IL-1β, and IL-17 signaling pathways. In mouse experiments, the deletion of NEDD4L led to the aggravation of inflammation induced by IL-17 and the aggravation of the symptoms of autoimmune encephalomyelitis (EAE), indicating that it plays an important anti-inflammatory and protective role in protecting the nervous system (12). In diabetic nephropathy (DN), Growth Differentiation Factor 15 (GDF-15) inhibits ubiquitin-dependent Inhibitor of Nuclear Factor Kappa B Kinase (IKK) degradation mediated by NEDD4L in podocytes, thus blocking the activation of the NF-κB signaling and reducing the inflammatory damage of diabetic nephropathy podocytes, which presents a pro-inflammatory effect of NEDD4L in this cell type (97). This is a key conflicting conclusion in current research: NEDD4L exerts an anti-inflammatory effect by inhibiting NF-κB signaling in most cell types, while it promotes NF-κB signaling activation in renal podocytes, and the underlying mechanism has not been fully elucidated, which may be related to the specific upstream regulatory signals and substrate expression profile in podocytes. The upstream receptor system can also regulate the activity of NEDD4L; the G protein-coupled receptor (GPCR) regulates the activity of E3 ligase by inducing the phosphorylation of the c-SRC-mediated NEDD4L tyrosine switch (Y485) in vascular endothelial cells, thus driving the activation of the TGF-beta Activated Kinase 1 Binding Protein 1 (TAB1)/p38 signaling pathway and exerting a pro-inflammatory effect, indicating that there are other regulatory control points in the inflammatory signal (98).In lung fibroblasts, NEDD4L restricts transforming growth factor β (TGF-β) signaling by degrading transforming growth factor β receptor II (TβRII). The TGF-β1/E2F4 axis is a key checkpoint that inhibits the progression of pulmonary fibrosis driven by NEDD4L, reflecting its anti-inflammatory and anti-fibrotic effects in this cell type (99). Collectively, the functional differences of NEDD4L in different cell types are determined by cell-specific substrate expression and upstream regulatory signals, and the lack of systematic verification of its bidirectional regulatory mechanism is a major limitation of current research.

ITCH extensively regulates a variety of signaling pathways, especially the c-Jun N-terminal Kinase (JNK) and NF-κB signaling, which are closely related to inflammation. Through its E3 ubiquitin ligase activity, ITCH targets key kinases and transcription factors, regulates the expression of inflammatory factors, and affects the fate of cells (85, 100, 101). The inflammatory regulatory function of ITCH exhibits significant cell type context dependence, which is the core driver of its bidirectional regulatory effects. For example, in epidermal growth factor (EGF) signaling in HEK 293T cells and primary hepatocytes, ITCH plays a pro-inflammatory role through JNK-dependent phosphorylation by degrading the anti-apoptotic protein c-FLIP and enhancing JNK pathway activation (102, 103). In contrast, in T cells(CD4^+^ T cells), ITCH is activated through JNK1-dependent phosphorylation, thereby promoting the polyubiquitination and degradation of transcription factors JunB and c-Jun, inhibiting Th2 and Th17 cell differentiation, which play an anti-inflammatory and immune tolerance maintenance role (104, 105). In intestinal epithelial cells and macrophages, ITCH can also inhibit the NF-κB signaling by mediating K63-linked ubiquitination of RIP2, reduce the transcriptional activity of pro-inflammatory factors, and further inhibit the occurrence of chronic inflammation (106, 107). In neuroinflammatory and immunomodulation, ITCH plays a key role in balancing cell apoptosis and inflammatory signaling in microglia and neurons. It can inhibit the continuous activation of inflammatory responses by promoting the degradation of ubiquitin-mediated inflammatory transcription factors, thus improving the pathological state of neuroinflammatory-related diseases (108). However, ITCH shows a complex bidirectional regulatory effect in the Nucleotide-Binding Oligomerization Domain Containing 2 (NOD2)/Receptor-Interacting Serine/Threonine Kinase 2 (RIP2) signaling pathway, and its anti-inflammatory or pro-inflammatory effect depends on the specific signaling pathway and cell environment. ITCH plays an anti-inflammatory function by inhibiting NF-κB signals and maintaining immune tolerance. However, in the MAPK pathway, it shows a local pro-inflammatory effect by activating JNK/p38, thereby regulating the activation state of immune cells and the secretion of inflammatory mediators. This multi-layered regulation makes ITCH a critical regulatory node in inflammatory responses (16, 101, 107). Notably, current studies have two key limitations: first, most studies only focus on its function in a single cell type, lacking a systematic comparison of its substrate preference and functional differences across multiple cell types; second, the *in vivo* functional verification mostly uses systemic knockout models, which cannot distinguish the contribution of ITCH in different cell types to the overall inflammatory phenotype, leading to the inability to clarify its core regulatory role in specific disease contexts.

As members of the NEDD4 subfamily, SMURF1 and SMURF2 mainly play a role by regulating the TGF-β signaling pathway in the inflammatory microenvironment. The TGF-β signaling pathway is a key pathway that regulates immune balance, fibrosis, and inflammatory processes (109–111). SMURF1/2 can target TGF-β receptors and their downstream effectors, such as Small Mothers Against Decapentaplegic (Smad) proteins, and promote their degradation through ubiquitination in renal tubular epithelial cells and lung fibroblasts, thus inhibiting the excessive activation of TGF-β signals, thereby regulating inflammatory responses and fibrosis process (112). In chronic inflammation and fibrotic diseases, SMURF1/2 help alleviate inflammation-related tissue damage and excessive fibrosis by limiting TGF-β signaling transduction (113). Moreover, SMURF1/2 also participate in the cross-talk of other inflammation-related signaling pathways, such as influencing extracellular matrix remodeling and immune cell migration, thereby regulating the composition and function of the inflammatory microenvironment (114, 115). Their ubiquitin ligase activity makes SMURF1/2 key molecules that bridge inflammation signals and tissue repair mechanisms. However, conflicting conclusions have been reported in existing studies: in retinal pigment epithelial cells, SMURF1 promotes inflammatory responses by enhancing the phosphorylation of NF-κB p65 and the activation of the NLRP3 inflammasome, exerting a pro-inflammatory effect (116); in T lymphocytes, SMURF2 enhances pro-inflammatory cytokine secretion by regulating the STAT3/JNK signaling pathway, also showing a pro-inflammatory function (111). The core reason for this functional discrepancy is the cell type context dependence: SMURF1/2 mainly exert anti-inflammatory and anti-fibrotic effects in tissue parenchymal cells by inhibiting the TGF-β pathway, while they play a pro-inflammatory role in immune cells and disease-specific parenchymal cells by regulating the NF-κB and MAPK pathways. Current research has notable limitations: most studies focus on their regulatory roles in fibrotic diseases, while their functions in acute inflammatory responses and autoimmune diseases are insufficiently studied; in addition, the substrate specificity and functional differences between SMURF1 and SMURF2 in the same cell type have not been clearly distinguished, leading to an ambiguous understanding of their independent regulatory roles.

As HECT-type E3 ubiquitin ligases, WWP1 and WWP2 affect inflammatory responses by regulating the stability of key signaling proteins. Notably, the expression of WWP2 significantly decreases in atherosclerosis models, and its overexpression can alleviate the damage to human umbilical vein endothelial cells (HUVECs) caused by ox-LDL, which is reflected by reduced oxidative stress and inflammatory responses. Research indicates that programmed cell death protein 4 (PDCD4) may be a substrate of WWP2, and WWP2 binds to PDCD4 and promotes its ubiquitin-mediated degradation, thereby activating the antioxidant enzyme Heme Oxygenase 1 (HO-1) pathway to alleviate endothelial damage (117). In cardiovascular diseases, WWP1 ubiquitination degrades connexin 43 (Cx43), which leads to left ventricular hypertrophy and fatal arrhythmia. WWP1 overexpression exacerbates inflammatory responses after myocardial infarction through the MAPK pathway. Although the role of WWP1 in the inflammatory process has yet to be explored, as a paralogous family member of WWP2, it may also play an important role in similar signaling pathways (118).

In summary, the NEDD4 family ligases exert dual regulatory roles as both a “timer” and “damper” of inflammatory signaling cascades. Its pro-inflammatory or anti-inflammatory effect depends on the specific signaling pathway and cell environment, with significant cell type context dependence. The core determinants of this context-dependent regulation include: first, the cell type-specific expression profile of target substrates, which leads to the preferential binding of NEDD4 family members to high-abundance substrates in different cells, thereby producing opposite inflammatory regulatory effects; second, the type of ubiquitin chain catalyzed in different cellular contexts, with K48-linked ubiquitination mostly leading to substrate degradation and anti-inflammatory effects, while K63/K33-linked ubiquitination mostly mediates signal activation and pro-inflammatory effects; third, the difference in upstream regulatory signals in distinct cell types, which affects the enzyme activity, subcellular localization, and substrate binding ability of NEDD4 family ligases. It may have the opposite inflammatory effect in different cells, and the conflicting conclusions in existing studies are mostly derived from the neglect of this cell type context dependence, which is a key issue to be resolved in future research.

### 3.3 Functional regulation of the NEDD4 subfamily in inflammatory cells

The NEDD4 subfamily plays a multi-level and multi-dimensional regulatory role in inflammatory cells, regulating inflammatory outcomes in a way that depends on cell type and pathway, and plays a particularly prominent role in macrophages and T cells. The role of NEDD4–1 in macrophages is mainly to regulate the phagocytic function of macrophages and the release of inflammatory mediators, thus affecting the inflammatory process (93). In atherosclerosis, NEDD4–1 reduces the inflammatory activation of macrophage-derived foam cells and inhibits the expression of SASP-related proteins by promoting the degradation of the ubiquitin-mediated GATA4-p62 complex. It is reported that Berberine promotes the ubiquitination degradation of GATA4-p62 complex by activating the RXRα/PPARγ complex, thus enhancing the E3 ligase activity of NEDD4-1, thereby inhibiting inflammatory responses of foam cells derived from macrophages and showing potential resistance. The effect of atherosclerosis (88). In acute lung injury (ALI) caused by exposure to phosgene, NEDD4–1 can prevent inflammation and oxidative stress of acute lung injury by promoting the ubiquitination degradation of Notch1 (89). In pulmonary ischemia-reperfusion injury (LIRI), the overexpression of NEDD4–1 can reduce ferroptosis and tissue damage through the ubiquitination degradation of the glutamine transporter SLC1A5 (ASCT2), and link ubiquitin signaling to the process of inflammatory cell death (119). In the bacterial-viral co-infection model, NEDD4–1 is involved in regulating the activation of NLRP3 inflammasome and the death associated with GSDMD, and further regulating the pathological process of inflammation (120).

ITCH is a member of the NEDD4 subfamily. In T cells, ITCH plays the role of activation threshold, differentiation determination, and immune tolerance. ITCH can inhibit the activation of abnormal T cells and sustain cellular immune tolerance, so it plays a protective role in autoimmune diseases and chronic inflammation (107, 121, 122). Although the specific mechanism needs further research, current research shows that ITCH is the key molecule that determines the fate of T cells, further affecting the intensity and duration of inflammatory responses (105, 121, 123).

Collectively, the NEDD4 subfamily regulates immune cell function at multiple levels - from macrophage inflammation output and cell programmatic death to T cell activation and differentiation, with significant functional heterogeneity across different cell types. For example, NEDD4–1 mainly exerts an anti-inflammatory effect by inhibiting inflammasome activation and pro-inflammatory cytokine secretion in macrophages, while ITCH plays a core role in maintaining immune tolerance by regulating T cell differentiation and inhibiting excessive adaptive immune activation; WWP2 mainly alleviates vascular inflammatory damage by regulating endothelial cell function, while SMURF1/2 are mainly involved in the regulation of fibrosis-related inflammation in fibroblasts. We have a more complete understanding of the role of the NEDD4 subfamily in inflammatory regulation. Therefore, the NEDD4 subfamily can provide pro-inflammatory or anti-inflammatory instructions according to the pathways and cellular environment involved. However, current research has notable limitations: first, most functional studies are limited to macrophages and CD4^+^ T cells, while the regulatory roles of NEDD4 subfamily members in neutrophils, B cells, innate lymphoid cells, and other immune cell subsets remain largely unelucidated; second, most *in vivo* studies use systemic knockout models, lacking cell type-specific knockout verification, which cannot distinguish the independent contribution of different cell types to the overall inflammatory phenotype; third, the research on HECW1/2, a member of the NEDD4 subfamily, is extremely scarce, and their regulatory roles in inflammation are almost blank, forming a significant research gap. In addition, they also provide a theoretical basis and potential targets for the development of new strategies for the treatment of inflammatory and immune-related diseases. **Table 2** summarizes the details of the NEDD4 family, including their substrates, interacting partners, cell types, inflammatory effects, related pathways, and functions in Inflammation, with additional supplementation of cell type-specific functional characteristics and conflicting research conclusions.

**Table 2:** NEDD4 subfamily: inflammatory characteristics and regulators.

NEDD4 subfamily	Substrate	Cell types	Inflammatory effect	Known linkage type	Pathway	Biological function	Reference
NEDD4-1	GATA4-p62	RAW264.7, PMs	Anti-	K48	RXRα/PPARγ/NEDD4-GATA4-p62	Cell growth, cell survival, autophagy, Innate immune, pyroptosis, inflammatory responses	(88)
	Notch1	AEC2s	Anti-	K48	NEDD4-Notch1		(89)
	TBK1	A549, PBMCs	Anti-	K27	NEDD4-TBK1-NDP52		(90)
	CSF1R	BMDMs, TAMs, THP-1	Anti-	K48	USP18-NEDD4-CSF1R		(91)
	SF3A2	THP-1, PMs, CMECs, H9c2	Anti-	K48	NEDD4-SF3A2-NLRP3		(93)
	NAMPT	THP-1	Anti-	K48	TLR4-NLRP3-eNAMPT		(94)
	APEX1	HUVECs	Anti-		NEDD4-APEX1		(95)
	SLC1A5	MLE-12, A549	Anti-	K48	NEDD4-SLC1A5		(119)
	NLRP3/GSDMD	BALB/c, A549	Anti-		NEDD4-NLRP3/GSDMD		(120)
	IκBα	HeLa, HEK-293T	Pro-	K48	TMEPAI-NEDD4-IκBα		(96)
NEDD4-2	MEKK2	MEFs, PPECs, astrocytes	Anti-	K27	NEDD4L-MEKK2	Apoptosis, growth arrest	(12)
	IKK/NF-κB	HPCs	Pro-		GDF-15-NEDD4L-IKK/NF-κB		(97)
	GPCR-c-Src	EA.hy926	Pro-		GPCR-c-Src-NEDD4-2-p38		(98)
	TβRII	lung fibroblasts, A549	Anti-		E2F4-Nedd4L-TβRII		(99)
ITCH	cFLIP	HepG2	Pro-		ATM-ITCH-cFLIP/Jun	DNA damage, Immune, inflammatory responses, cell growth, cell proliferation	(100)
	UbcH7	HEK 293T	Pro-		IKKβ-ITCH-Ser687-UbcH7		(101)
	EGF-JNK	HEK 293T	Pro-		EGF-JNK-ITCH		(102)
	FMN	HEK 293T	Anti-		FMN-ITCH		(102)
	c-FLIPL/JNK	primary hepatocytes, embryonic fibroblasts	Pro-	K48	JNK1-ITCH-c-FLIPL		(103)
	JunB/c-Jun	CD4+T (Th2)	Anti-	K48	JNK1-ITCH-JunB/c-Jun		(104)
	IKK	Jurkat T, A549	Pro-		IKK-ITCH-TNF		(106)
	Foxp3	CD4+T(Treg)	Anti-	K27	ITCH-TIEG1-Foxp3		(16, 121)
	A20/Cyld	HEK 293T	Anti-	K48	ITCH-A20/Cyld		(16)
	RIP2	HT-29, BMDMs	Anti-	K63	ITCH-RIP2-NF-κB		(107)
	RORγt	CD4+T (Th17)	Anti-		ITCH-RORγt		(105)
	SHP-1	CD4+T (Th2)	Anti-	K27/K29	ITCH-WWP2-SHP-1		(123)
SMURF1/SMURF2	TGF-β/Smad	renal tubular epithelial cells, renal fibroblasts	Anti-	K48	Smurfs-TGF-β/Smad	Immune, cell polarity, cell migration, cell proliferation, cell senescence, inflammatory responses	(114)
	m6A-YTHDF2	MC3T3-E1	Anti-		m6A-YTHDF2-Smad7/Smurf1		(124)
	TGF-β1	ARPE-19	Pro-		Smurf1-TGF-β1-NF-κB		(116)
	STAT3/JNK	PBL T-cells, Hut78 T cells	Pro-		STAT3/JNK-SMAD7/SMURF2/SKI		(111)
	SMAD7	HaCaT, 293, COS-1	Anti-	K48	CD109-SMAD7-Smurf2		(112)
	TTC3	BEAS-2B, NHLFs	Anti-	K48	TTC3-SMURF2-SMAD2/3		(113)
WWP1	MAPK	Cardiomyocytes, Cardiac Fibroblasts	Pro-		WWP1-MAPK	Inflammatory responses	(118)
WWP2	PARP1	293T, H9c2	Anti-	K29/K48	WWP2-PARP1	oxidative stress, inflammatory responses, apoptosis	(14)
	PDCD4	HUVECs, ApoE-	Anti-		WWP2-PDCD4-HO-1		(117)
	BRCC3	BMDMs	Anti-	K48/K63	ABRO1-WWP2-BRCC3		(125)

## 4 Roles of other HECT-type E3 ubiquitin ligases in inflammation

### 4.1 Regulatory mechanisms of HUWE1 in inflammation

HUWE1 is a large HECT-type E3 ubiquitin ligase with a unique circular substrate binding structure and a wide substrate spectrum, which can regulate stress response, proliferation, apoptosis, and signal transduction. Recent studies show that HUWE1 can regulate a variety of inflammatory signaling molecules, mainly NF-κB and NLRP3, which in turn participate in the activation of inflammasomes and affect the immune response of cells, with significant cell type context dependence and conflicting research conclusions. In macrophages, HUWE1 activates NLRP3, Absent In Melanoma 2 (AIM2), and NLRC4 inflammasomes by K27 ubiquitination to promote the release of IL-1β to resist bacterial infection, exerting a significant pro-inflammatory effect. In contrast, in sickle cell disease lung tissue cells, HUWE1 can degrade NF-κB p65 and E26 Transformation-Specific 1 (Ets-1) (inhibit Treg differentiation) to reduce excessive lung inflammation (126, 127). In retinal pigment epithelial cells and vascular endothelial cells, HUWE1 also enhances the expression of pro-inflammatory factors by promoting the activation of the NF-κB signaling, showing a pro-inflammatory effect (128, 129). This bidirectional regulation is mainly driven by cell type-specific substrate preference: in myeloid immune cells, HUWE1 preferentially targets NLRP3 and TRAF6 to promote inflammatory activation, while in parenchymal cells, it can directly degrade the p65 subunit of NF-κB to inhibit inflammatory responses. HUWE1 ubiquitinates NLRP3 to regulate the assembly and activation of inflammasomes, thus affecting the production of IL-1β and playing a major role in cells (130–132). This regulatory mechanism works not only in acute inflammation, but also in chronic inflammation and autoimmune diseases. However, current research has key limitations: due to the wide substrate spectrum of HUWE1, most studies only focus on a single substrate and pathway, lacking a systematic analysis of its substrate selection priority in different cell types; in addition, the *in vivo* functional verification mostly uses systemic knockout models, and the embryonic lethality of HUWE1 full knockout limits the in-depth study of its inflammatory regulatory function in adult animals, which is a major bottleneck in current research.

HUWE1 is also associated with death and other forms of inflammatory cell death. Pyroptosis is characterized by the release of inflammatory mediators to exacerbate inflammation. In atherosclerosis, circular RNA (circ_HUWE1) from the HUWE1 gene reduces the expression of inflammatory factors by regulating lipid metabolism and macrophage infiltration, which shows that circ_HUWE1 has anti-inflammatory effects and can further become a new therapeutic target for chronic inflammatory diseases (133). Accumulating evidence indicates that HUWE1 is mainly a positive regulator of inflammatory responses through the ubiquitin-mediated protein degradation pathway.

### 4.2 Association of E6AP with inflammatory response

E6-Associated Protein ((E6AP, also known as UBE3A) is a typical HECT-type E3 ubiquitin ligase, which plays a key role in inflammatory responses. E6AP has a clear role in cell cycle protein and transcription regulation, and also affects the proliferation and activation of immune cells. By ubiquitinating key transcription factors, cell cycle regulatory factors, and immune signaling intermediates, E6AP can affect the proliferation and activation of immune cells, thus affecting inflammation cascades (134–136). E6AP-mediated ubiquitination also governs autoimmune-related transcription factors, affecting cellular immune tolerance and activation, and providing a new perspective for understanding immune disorders (137).

The effect of E6AP on inflammation is mainly characterized as anti-inflammatory in HPV infection-related studies, while conflicting pro-inflammatory regulatory effects have been reported in other disease contexts, with significant cell type context dependence. In HPV16-related immune escape in HPV16-infected cervical epithelial cells, human papillomavirus 16 (HPV16) E6 cancer protein targets the N-terminal of IL-1β through the E6AP/p53 axis to promote ubiquitin-dependent IL-1β degradation, thus blocking the activation of the inflammasome. HPV16 E6 can also interfere with IRF3 function and inhibit the production of IFN-β, which depends on the activity of E6AP ligase, reflecting the anti-inflammatory effect of E6AP in this specific context (135, 138). Clinically, the expression of IL-1β decreased from cervical intraepithelial neoplastic lesions (CIN) to invasive carcinoma, which is related to the post-translational silence mechanism of E6AP (135). Additionally, E6AP indirectly inhibits the NF-κB signaling by ubiquitinating and degrading TNF Receptor Associated Factor 2 (TRAF2), a member of the tumor necrosis factor receptor-associated factor (TRAF) family; however, the precise mechanism remains incompletely elucidated and is currently controversial. In contrast, in macrophages, E6AP regulates the PGRN-C/EBPα-IL-10 axis by ubiquitinating and degrading the transcription factor C/EBPα, thereby suppressing IL-10, exerting a significant pro-inflammatory effect. Progranulin (PGRN) and E6AP maintain a mutually inhibitory dynamic equilibrium. In the sepsis model, the lack of PGRN will destroy this balance, which will lead to uncontrolled inflammation (134, 136–139). The core reason for this functional discrepancy is the cell type context dependence and the presence or absence of HPV E6 protein: in HPV-infected epithelial cells, E6AP is recruited by E6 to target anti-viral and pro-inflammatory substrates for degradation, exerting an anti-inflammatory effect; in physiological states without HPV infection, E6AP in macrophages mainly targets the anti-inflammatory transcription factor C/EBPα for degradation, playing a pro-inflammatory role. In general, current evidence cannot simply define E6AP as a key mediator that plays a major anti-inflammatory role in inflammation regulation, and its function is highly dependent on the cellular context and disease microenvironment. Current research has notable limitations: most studies focus on its role in HPV infection, while its physiological inflammatory regulatory function in uninfected states is insufficiently studied; in addition, its regulatory roles in adaptive immune T/B cells and other immune cells remain largely unelucidated.

### 4.3 Inflammatory regulatory functions of other HECT members

HECT Domain And Ankyrin Repeat Containing E3 Ubiquitin Protein Ligase 1 (HACE1) plays an important role in neurodegenerative diseases. HACE1 regulates oxidative stress, autophagy, and inflammation by governing the ubiquitination and stability of substrates such as Rac1 and OPTN and the stability of Nrf2, and participates in neurodegeneration and inflammatory conditions. As a regulating factor of the Tumor Necrosis Factor Receptor 1 (TNFR1) signal, HACE1 balances cell apoptosis and necroptosis. Deficiency of HACE1 leads to excessive inflammation activation and cancer. HACE1 plays an anti-inflammatory role by regulating the TNFR-NF-κB signaling, while activating the Nrf2 pathway to reduce oxidative stress, thus indirectly inhibiting inflammation. In summary, HACE1 may negatively regulate inflammation by regulating Ras-Related C3 Botulinum Toxin Substrate 1 (Rac1), TNFR-NF-κB, and Nuclear Factor Erythroid 2-Related Factor 2 (Nrf2) pathways, providing a theoretical basis for anti-inflammatory treatment (140–142).

Thyroid Hormone Receptor Interactor 12 (TRIP12), as an E3 ubiquitin ligase of the HECT-type E3 ubiquitin ligase superfamily, exerts tissue-specific bidirectional effects in inflammation by mediating substrate ubiquitination and pathway regulation. In inflammasome regulation, TRIP12(TRIPC) synergizes with Apoptosis-Resistance E3 Ubiquitin Protein Ligase 1 (AREL1) and Ubiquitin Conjugating Enzyme E2 L3 (UBE2L3) to modify specific sites on pro-IL-1β, promoting its proteasomal degradation and thereby reducing IL-1β release, which suppresses neutrophil inflammation (143). In neuroinflammation, TRIP12 degrades Methyltransferase Like 3 (METTL3) through K48-linked ubiquitination; deficiency of TRIP12 exacerbates neuroinflammation (144). In hepatic inflammation, chronic mild iron overload promotes TRIP12-YY1 interaction, accelerating Yin Yang 1 (YY1) degradation through K63-linked ubiquitination. This downregulates MicroRNA-122 (miR-122) and releases its inhibition on C-C Motif Chemokine Ligand 2 (CCL2), triggering inflammation independently of oxidative stress (145). In age-related inflammation, TRIP12 promotes ubiquitination and Sirtuin 7 (SIRT7) degradation, and activates NF-κB/CEBPβ signaling. Knocking out TRIP12 can restore SIRT7 levels and reduce inflammation (146). Collectively, these findings establish that TRIP12 is a multisubstrate hub with therapeutic relevance.

HECT Domain E3 Ubiquitin Protein Ligase 1 (HECTD1) regulates inflammation through a variety of substrates that rely on ubiquitination. HECTD1 and Ribosomal Protein S3 (Rps3) form a complex, which promotes Inhibitor Of Nuclear Factor Kappa B Alpha (IκBα) ubiquitination degradation, activates NF-κB, and aggravates colitis (147). HECTD1-mediated Forkhead Box F1 (FOXF1) degradation enhances inflammatory responses in lung injury. Among them, the small molecule TanFe blocks their interaction and stabilizes FOXF1, improving pulmonary vascular inflammation and barrier function (148). In osteoarthritis, promoter methylation downregulates the expression of HECTD1, prevents the ubiquitin-mediated degradation of Aurora Kinase A (AURKA), thereby promoting the translation of ADAM Metallopeptidase With Thrombospondin Type 1 Motif 12 (ADAMTS12) and exacerbating matrix decomposition and inflammation (149). HECTD1 can also inhibit the PI3K-Akt pathway by degrading Insulin Receptor Substrate 1 (IRS1), driving macrophage senescence and proinflammatory cytokine secretion. α-Klotho inhibits HECTD1 activity, which can reverse inflammation and vascular damage in diabetic retinopathy (150).

HECT Domain E3 Ubiquitin Protein Ligase 2 (HECTD2) ubiquitinates the anti-inflammatory protein Protein Inhibitor Of Activated STAT 1 (PIAS1) at the K48-linked site for proteasomal degradation, and is enhanced by the phosphorylation of PIAS1 mediated by Glycogen Synthase Kinase 3 Beta (GSK3β). The HECTD2 A19P polymorphism can reduce inflammatory responses, and the inhibitor BC-1382 has anti-inflammatory potential. In renal cell carcinoma, HECTD2 ubiquitinates and degrades Euchromatic Histone Lysine Methyltransferase 2 (EHMT2), which alleviates the inhibition of TNF Alpha Induced Protein 1 (TNFAIP1) and activates the pro-inflammatory p38/JNK pathway (151, 152). Collectively, HECTD2 plays a key pro-inflammatory role in the microenvironment of inflammatory diseases and tumor inflammation, and acts as an inflammatory driving factor for inflammatory diseases and tumor-related inflammation.

HECT Domain E3 Ubiquitin Protein Ligase 3 (HECTD3) exerts bidirectional regulation of inflammatory responses through ubiquitin modification or non-enzymatic pathways, which is the most typical member of the HECT-type E3 ubiquitin ligase superfamily with conflicting research conclusions, and its inflammatory function is completely dependent on cell type and pathological context. In diabetes-associated cognitive impairment neurons, HECTD3 stabilizes Mucosa Associated Lymphoid Tissue Lymphoma Translocation 1 (MALT1) by K63 ubiquitination, activates the JNK/c-JUN pathway, promotes NLRP3 inflammasome activation and neuronal pyroptosis, intensifying neuroinflammation, and exerts a significant pro-inflammatory effect (153). In contrast, in bone marrow-derived macrophages of gouty arthritis models, HECTD3 binds to the NACHT/LRR domain of NLRP3 via its DOC domain, inhibiting NLRP3-NIMA Related Kinase 7 (NEK7) interaction and inflammasome assembly. Its anti-inflammatory effect is independent of E3 ligase activity (154). During RNA viral infection in macrophages, HECTD3 modifies Protein Kinase R (PKR) with K33-linked ubiquitination, exhibiting dual regulation by both suppressing its antiviral activity and activating the NF-κB signaling to promote inflammatory cytokine release (155). In cardiomyocytes with myocardial hypertrophy, HECTD3 degrades Small Ubiquitin Like Modifier 2 (SUMO2) and Signal Transducer And Activator Of Transcription 1 (STAT1) through ubiquitination, which inhibits calcium-NAFT signaling and interferon (IFN)-mediated inflammation, respectively, and plays a protective protective and anti-inflammatory role (156). In liver sinusoidal endothelial cells during liver transplant ischemia-reperfusion injury, downregulation of HECTD3 suppresses K63-linked ubiquitination of TNF Receptor Associated Factor 3 (TRAF3), blocks the NF-κB signaling, and reduces inflammation during hypothermic oxygenated perfusion (HOPE), reflecting its pro-inflammatory effect in this cell type (157). The core reasons for this extreme functional heterogeneity include: first, cell type-specific substrate expression, which leads to HECTD3 targeting different substrates in distinct cell types; second, the separation of enzymatic and non-enzymatic functions, with its pro-inflammatory effect mostly dependent on its HECT-type E3 ubiquitin ligase activity, while its anti-inflammatory effect is mostly mediated by non-enzymatic protein-protein interactions; third, the difference in upstream activation signals in different pathological contexts, which determines the dominant functional mode of HECTD3. In a word, HECTD3 exhibits strict cell type context-dependent regulation of inflammatory signaling axes and maintains the inflammatory homeostatic state. However, current research has key limitations: no study has simultaneously compared the functional differences of HECTD3 in the same inflammatory model across different cell types, and the switch mechanism between its enzymatic and non-enzymatic functions has not been elucidated, leading to the inability to define its core regulatory role in specific inflammatory diseases.

In summary, these “other” HECT-type E3 ligases emphasize that ubiquitin-mediated inflammatory signal transduction is not a linear process, but a complex regulatory network. This provides conditions for a comprehensive analysis of the mechanism of HECT-type E3 ligase regulating NF-κB activation, inflammasome assembly, and downstream cytokine transcription procedures. **Table 3** summarizes the details of the other HECTs, including their substrates, interacting partners, cell types, inflammatory effects, related pathways, and functions in Inflammation.

**Table 3:** Other HECT-type E3 ubiquitin ligases: inflammatory characteristics and regulators.

Other HECTs	Substrate	Cell types	Inflammatory effect	Known linkage type	Pathway	Biological function	Reference
HUWE1	NF-κB p65	sickle cell disease	Anti-		HUWE1-NF-κB p65	Innate immune, inflammatory responses, apoptosis	(126, 127)
	NLRP3	BMDMs, THP-1/PBMCs	Pro-	K27	HUWE1-NLRP3		(126, 127)
	TRAF6	HEK293T, U2OS, 293F	Pro-	K48/K63	HUWE1-TRAF6-NF-κB p65		(128)
	BDB	HUVECs, RAW264.7	Pro-		HUWE1-BDB-NF-κB p65		(129)
UBE3A(E6AP)	C/EBPα	BMDMs, RAW264.7	Pro-	K48	PGRN-E6AP-C/EBPα-IL-10	Innate immune, inflammatory responses, apoptosis	(134)
	p53	immE6/E7	Pro-	K48	16E6-E6AP-p53-pro-IL-1β		(135, 136)
	IFN-κ	CIN612-9E, HeLa	Pro-		E6AP-p53-IFN-κ		(139)
HACE1	Rac1	BV2, SH-SY5Y, striatal neurons	Anti-		HACE1-Rac1-JAK2-STAT1	Oxidative stress, autophagy, inflammation, neuroinflammation	(140)
	TNFR1	MEFs, dopaminergic neurons	Anti-	K63	HACE1-TNFR1-NF-κB		(141)
	Nrf2	striatal neurons, cortical neurons, U87/SF295	Anti-		HACE1-Nrf2		(140)
	OPTN	striatal neurons	Anti-		HACE1-OPTN		(140)
TRIPC(TRIP12)	SIRT7	IMR90	Pro-		SIRT7-TRIP12-NUCKS1	DNA damage, DNA Repair, cell cycle progression, inflammatory responses	(146)
	YY1	L02	Pro-	K48	TRIP12-YY1		(145)
	METTL3	BV2	Anti-	K48	TRIP12-METTL3-M6a-BATF		(144)
	pro-IL-1β	BMDMs	Anti-	K27/K29/K33	UBE2L3-TRIP12/AREL1-pro-IL-1β		(143)
HECTD1	IκBα	HIEC, HCT116	Pro-	K48	HECTD1-Rps3-IκBα	Embryogenesis, DNA damage repair, inflammatory responses	(147)
	IRS1	PBMCs, RAW264.7	Pro-	K48	HECTD1-IRS1-PI3K/Akt		(150)
	AURKA	Primary Chondrocytes	Anti-	K48	HECTD1-AURKA-ADAMTS12		(149)
	FOXF1	Ecs, MFLM-91U	Pro-	K48	TanFe-HECTD1-FOXF1		(148)
HECTD2	PIAS1	MLE, U937	Pro-	K48	HECTD2-PIAS1	Innate immune, inflammatory responses	(151)
	EHMT2	Caki-1, ACHN, A498, HK2	Pro-	K48	HECTD2-EHMT2-TNFAIP1-p38/JNK		(152)
HECTD3	PKR	BMDMs, RAW264.7,	Pro-	K33	HECTD3-PKR	Apoptosis, pyroptosis, neuroinflammation, oxidative stress	(155)
	TRAF3	BRL-3A, LSECs	Pro-	K63	HECTD3-TRAF3- NF-κB		(157)
	MALT1	rHNs, PC12	Pro-	K63	HECTD3-MALT1-JNK-NLRP3		(153)
	NLRP3	BMDMs	Anti-		HECTD3-NLRP3-NEK7-ASC		(154)

## 5 Integration of inflammatory signaling networks mediated by HECT-type E3 ubiquitin ligases

**Figure 2:**
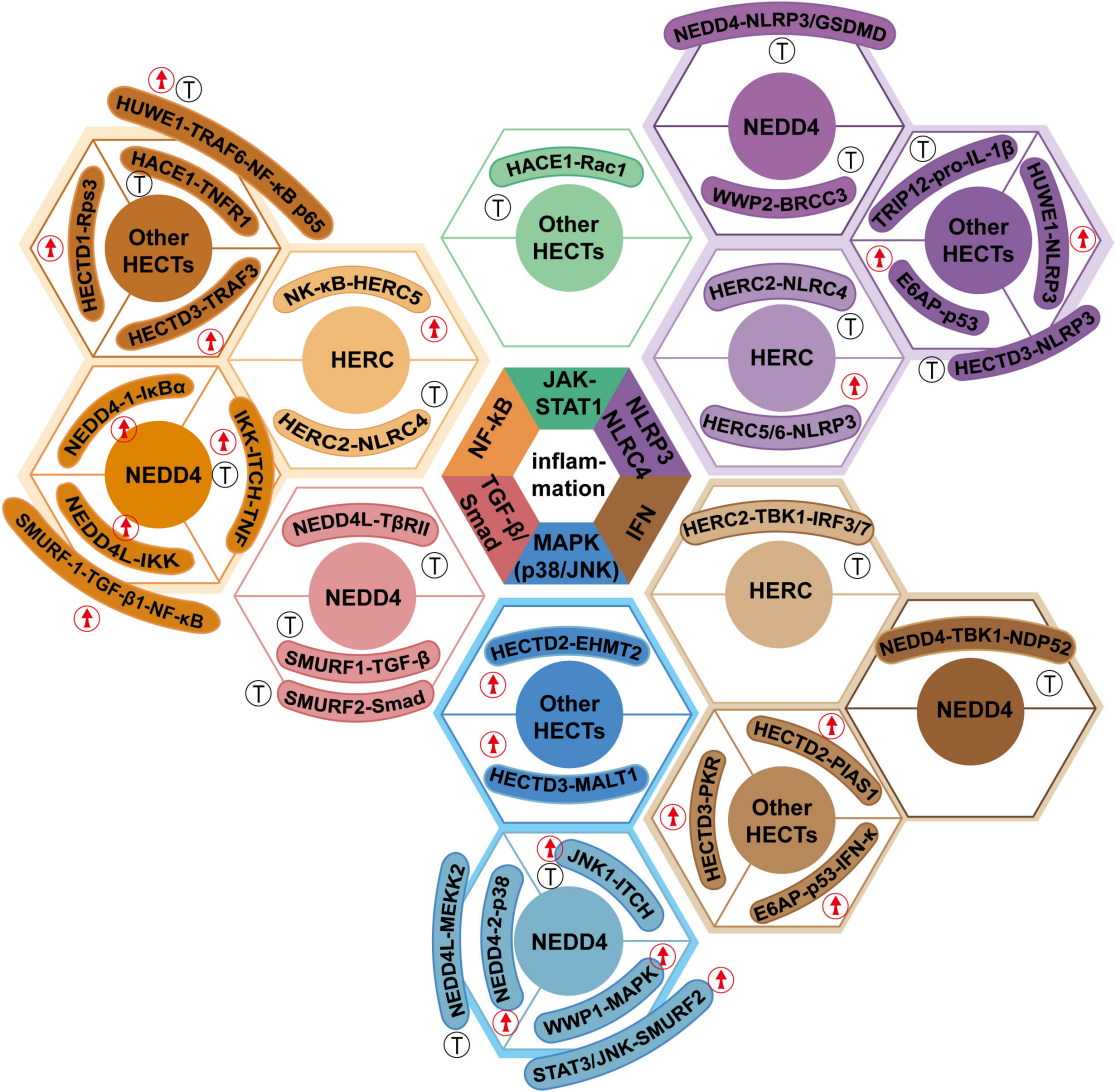
Integrated mechanistic model of HECT-type E3 ubiquitin ligases orchestrating inflammatory responses via core signaling pathways. This schematic summarizes the collective and context-dependent regulation of major inflammatory signaling cascades (NF-κB, MAPK, NLRP3/NLRC4/AIM2 inflammasomes, and type I interferon signaling) by HECT-type E3 ligase subfamilies (HERC, NEDD4) and other key HECT members (HUWE1, E6AP, HECTD3). Distinct HECT members exert pro-inflammatory or anti-inflammatory effects through differential ubiquitination modifications and ISGylation, with cell type-specific substrate targeting and ubiquitin chain linkage specificity as the core determinants of their functional heterogeneity. Arrows indicate activation/promotion of signaling, and blunt ends indicate inhibition/suppression; different colors distinguish HECT subfamilies for visual clarity.

A central mechanistic model depicting the collective regulation of major inflammatory signaling pathways by HECT-type E3 ligases is summarized in **Figure 2**, which integrates the divergent functions of HERC, NEDD4 and other HECT members in NF-κB, MAPK, inflammasome and interferon signaling. This schematic illustrates the regulatory mechanisms of HECT-type E3 ubiquitin ligases, providing a unified framework for understanding HECT-mediated inflammatory signaling networks.

### 5.1 Role of ubiquitination in the NF-κB signaling

The NF-κB signaling is a key regulatory pathway closely associated with activation and ubiquitination. HECT-type E3 ubiquitin ligases regulate the activity of the NF-κB signaling by ubiquitinating the IKK complex and its downstream effectors, thereby controlling the expression of inflammation-related genes. Ubiquitination not only promotes protein degradation but also acts as a signaling scaffold, determining the magnitude and duration of the pathway through distinct ubiquitin chain structures (158–160).

In the downstream of the NF-κB signaling, HERC5 participates in the progression of esophageal squamous cell carcinoma through the regulation of circJPH1. By binding to XRCC6, circJPH1 promotes the nuclear translocation of XRCC6 and activates the NF-κB signaling. This activation subsequently upregulates HERC5 expression and promotes tumor cell proliferation and migration, indicating that HECT-type E3 ligases can drive inflammation and tumor progression by regulating the NF-κB signaling in the disease microenvironment (63).

NEDD4–1 promotes ubiquitin-dependent IκBα degradation via Transmembrane Prostate Androgen Induced Protein 1 (TMEPAI), thereby reducing the inhibitory effect of IκBα on NF-κB. This enhances the activity of the NF-κB signaling and promotes cell proliferation and tumor development. This mechanism demonstrates that HECT-type E3 ubiquitin ligases not only regulate upstream kinases but also act directly on inhibitors of the NF-κB signaling to amplify signaling (96).

As a member of HECT-type E3 ubiquitin ligases, NEDD4L has been shown to inhibit IL-17 receptor-mediated signal transduction via K48-linked polyubiquitination-dependent degradation of MEKK2. This effect weakens the activation of p38 and NF-κB, reduces the production of pro-inflammatory cytokines, and thus confirms its role as a negative regulator in inflammatory responses (12). This polyubiquitination-mediated degradation mechanism reveals that HECT-type E3 ubiquitin ligases indirectly regulate the activity of the NF-κB signaling by modulating the stability of upstream kinases (116, 124, 161).

SMURF1 is another E3 ubiquitin ligase containing C2-WW-HECT domains, and its expression is upregulated in retinal degenerative disease models. Studies have demonstrated that SMURF1 promotes inflammatory responses by regulating the phosphorylation status of NF-κB and the expression of the NLRP3 inflammasome (162). The interaction between SMURF1 and Beta-Transducin Repeat Containing E3 Ubiquitin Protein Ligase (β-TrCP) affects the ubiquitination-dependent degradation of IκBα. β-TrCP is an F-box E3 ubiquitin ligase that mediates the ubiquitin-dependent degradation of IκBα, thereby triggering NF-κB activation. By maintaining the stability of β-TrCP, SMURF1 indirectly regulates the NF-κB signaling, reflecting the synergy between HECT-type E3 ubiquitin ligases and other ubiquitin ligases (163–165).

TRIP12 and Ubiquitin Protein Ligase E3 Component N-Recognin 5 (UBR5) promote the degradation of the deubiquitinase OTU Deubiquitinase 5 (OTUD5), which affects TNF-α-induced NF-κB signal transduction. This highlights the multi-layered regulatory mechanism of HECT-type E3 ubiquitin ligases in the NF-κB signaling via complex ubiquitination signaling (166).

Linear ubiquitination is primarily mediated by Linear Ubiquitin Chain Assembly Complex (LUBAC), which plays a critical role in the assembly and activation of the IKK complex and protects cells from excessive apoptosis during inflammation (167–169). Although HECT-type E3 ubiquitin ligases do not directly form linear ubiquitin chains, they regulate the NF-κB signaling by modulating the ubiquitination status of relevant substrates, thereby cooperating with the linear ubiquitination mechanism.

Collectively, distinct members of the HECT-type E3 ubiquitin ligase superfamily exhibit synergistic and antagonistic effects in the NF-κB signaling: HERC5 participates in tumor-related signal activation; TMEPAI, as a recruiter, facilitates NEDD4-1-mediated IκBα degradation; NEDD4L inhibits signal transduction by promoting kinase degradation; SMURF1 enhances the expression of pro-inflammatory factors, and TRIP12 and UBR5 regulate the stability of deubiquitinases. These examples illustrate the diversity and complexity of HECT-type E3 ubiquitin ligases in regulating NF-κB signaling (12, 96, 166, 170, 171). This multi-faceted regulatory mechanism ensures the fine-tuning of inflammatory responses, preventing tissue damage caused by excessive activation, and provides a theoretical basis for the targeted therapy of distinct HECT family members (172–174).

### 5.2 Regulation of the inflammasome by HECT-type E3 ubiquitin ligases

HECT-type E3 ubiquitin ligases play key roles in regulating inflammasomes, and exert critical regulatory functions in the assembly and activation of the NLRP3 inflammasome in particular. The NLRP3 inflammasome is a central component of the innate immune system. Its activation drives the maturation and release of proinflammatory cytokines such as IL-1β and induces inflammatory cell death (125, 153, 175, 176). HECT-type E3 ubiquitin ligases modulate the ubiquitination of NLRP3 and its associated proteins, thereby influencing the assembly efficiency and activation status of the inflammasome and shaping the magnitude and duration of immune responses (125, 153, 177).

HERC5 and HERC6 target the NLRP3 protein via ISGylation modification, enhancing its stability and promoting inflammasome activation. Under conditions of viral infection and type I interferon stimulation, HERC5/6-mediated NLRP3 ISGylation inhibits its K48-linked ubiquitination and degradation, thereby sustaining NLRP3 activity (24).

WWP2 indirectly inhibits NLRP3 inflammasome activation by regulating the stability of the deubiquitinase BRCC3. As a lysine 63-specific deubiquitinase, BRCC3 stability is negatively regulated by WWP2-mediated ubiquitination and degradation. Upon WWP2 overexpression, BRCC3 levels decrease, and NLRP3 inflammasome activation is inhibited. These findings indicate that HECT-type E3 ubiquitin ligases can regulate inflammasome-mediated immune responses by modulating the stability of deubiquitinases (125).

HECTD3 has been shown to activate the JNK signaling pathway by stabilizing MALT1, thereby promoting NLRP3 inflammasome activation and inflammatory activity, which exacerbates diabetes-related cognitive dysfunction. HECTD3 knockout significantly suppresses the expression of NLRP3 and Cysteine Aspartic Protease 1 (caspase-1), reduces GSDMD cleavage, and diminishes the release of proinflammatory cytokines IL-1β and IL-18, thus protecting neuronal survival and improving cognitive function (153).

Conversely, HECTD3 inhibits NLRP3 inflammasome assembly and activation by blocking the interaction between NLRP3 and NEK7. Notably, this function operates independently of its E3 ligase activity. The DOC domain of HECTD3 interacts with the NACHT/LRR domains of NLRP3, thereby impeding NEK7-mediated NLRP3 oligomerization. This effectively inhibits IL-1β secretion and subsequent inflammatory responses, highlighting the multifaceted mechanisms by which HECT-type E3 ubiquitin ligases regulate inflammation (154).

Moreover, HECT-type E3 ubiquitin ligases regulate the pyroptosis mediated by inflammasomes through ubiquitination. Ubiquitination not only affects the stability of NLRP3 protein but also regulates the activity of downstream key effector molecules such as caspase-1 and GSDMD, thereby controlling the initiation and progression of pyroptosis (153, 178). Studies have found that HECT-type E3 ubiquitin ligases subfamily members TRIP12 and AREL1 promote the degradation of pro-IL-1β by adding specific types of polyubiquitin chains, specifically K27-, K29-, and K33-linked chains. This limits the production of mature IL-1β and thus inhibits inflammatory responses, revealing the important role of HECT-type E3 ubiquitin ligases in balancing inflammatory response regulation (143).

Finally, the regulatory role of HECT-type E3 ubiquitin ligases also provides new ideas for the treatment of inflammation-related diseases. For example, the small molecule inhibitor M01, targeting HECT-type E3 ubiquitin ligases, alleviates neuroinflammation and cell death and improves cognitive and visual functions in models of Alzheimer’s disease and retinal ischemic injury by inhibiting the activation of the NLRP3 inflammasome (179).

In summary, HECT-type E3 ubiquitin ligases regulate the assembly, activation, and inflammasome-mediated pyroptosis of NLRP3 inflammasomes via diverse enzymatic and non-enzymatic mechanisms. This deepens the mechanistic understanding of NLRP3 inflammasome regulation and offers novel therapeutic targets for the intervention against inflammasome-driven inflammatory diseases (177).

### 5.3 Ubiquitination and cytokine expression regulation

HECT-type E3 ubiquitin ligases affect the cytokine program by controlling the ubiquitination of key transcription factors and signaling intermediates, thereby regulating the production of inflammatory mediators such as IL-1β and TNF-α (180). As an important post-translational modification, ubiquitination can dynamically regulate the activity of signaling pathways, thereby controlling the initiation and termination of inflammatory responses (52, 181).

HECT-type E3 ubiquitin ligases regulate the dynamic balance of inflammatory responses through ubiquitination, thereby ensuring the timely initiation and effective resolution of inflammation (108, 182, 183). HERC2 catalyzes K33-linked ubiquitination of TBK1, promotes type I interferon production, enhances antiviral immune responses, and highlights the functional diversity of HECT family members in inflammatory and immune regulation (37). In addition, this mechanism emphasizes that ubiquitination not only serves as a degradation signal but also acts as a regulatory switch for signal activation, participating in the amplification and integration of inflammatory signaling cascades (184).

In the NEDD4 subfamily, studies have shown that the member NEDD4L inhibits IL-17 receptor signaling by mediating the ubiquitin-dependent degradation of MEKK2, thereby reducing the expression of pro-inflammatory cytokines such as TNF-α and IL-1β, reflecting a negative regulatory mechanism in inflammation (12). As an activator of NEDD4 family E3 ubiquitin ligases, Nedd4 Family Interacting Protein 1 (Ndfip1) interacts with NEDD4–1 or ITCH to regulate the NF-κB signaling, which limits the production of pro-inflammatory cytokines and highlights the role of HECT-type E3 ubiquitin ligases in shaping inflammatory signaling (185).

In vascular inflammation, the HECT-type E3 ubiquitin ligase WWP2 degrades PDCD4 via ubiquitination, activates the HO-1 antioxidant pathway, reduces oxidative stress and inflammation, prevents endothelial injury and the progression of atherosclerosis, and exerts a protective role in the inflammatory environment (117). In neuroinflammation, TRIP12 promotes the K48 ubiquitination and degradation of METTL3 and inhibits activation of the METTL3-BATF axis, thereby reducing the expression of inflammatory factors such as TNF-α and alleviating inflammatory responses and neurotoxicity following brain injury (144). These examples demonstrate that HECT-type E3 ubiquitin ligases regulate not only canonical inflammatory signaling pathways but also the transcriptional and epigenetic networks that govern cytokine secretion (180, 183).

In summary, HECT-type E3 ubiquitin ligases form a complex, dynamically regulated network of inflammatory responses by mediating the ubiquitination of transcription factors (such as NF-κB, BATF), signaling molecules (such as MEKK2, TBK1), and precursors of inflammatory mediators. Ubiquitination not only modulates the synthesis and secretion of inflammatory factors such as IL-1β and TNF-α but also maintains the spatiotemporal balance of inflammatory responses by regulating the activation and resolution of signaling pathways, thereby preventing tissue damage caused by uncontrolled inflammation. The inflammatory effects of most identified HECT-type E3 ubiquitin ligases are illustrated in **Figure 2**.

### 5.4 Cell type-specific regulatory functions of HECT-type E3 ubiquitin ligases in inflammation

Accumulating evidence demonstrates that HECT-type E3 ubiquitin ligases exert distinct, even opposite, inflammatory regulatory effects in the four core cell subsets driving inflammatory responses: macrophages, dendritic cells (DCs), T lymphocytes, and epithelial/endothelial cells (13, 186). This section systematically integrates the cell type-specific regulatory rules of all HECT subfamilies, and clarifies the core molecular mechanisms underlying their functional heterogeneity, fully addressing the unelucidated cell type context dependence in existing studies.

Macrophages: As the core effector cells of innate immunity, macrophages are the main cell type where HECT-type E3 ubiquitin ligases exert inflammatory regulatory functions. In this cell subset, the NEDD4 subfamily members (NEDD4-1, ITCH, WWP2), HERC2, HACE1 and TRIP12 mainly form an anti-inflammatory regulatory axis, by targeting key components of the NF-κB and NLRP3 pathways for ubiquitination degradation, thereby inhibiting macrophage inflammatory polarization, inflammasome activation and pro-inflammatory cytokine secretion (107, 125, 141). In contrast, HERC5/6, HUWE1, HECTD1/2 mainly form a pro-inflammatory regulatory axis, by stabilizing NLRP3 via ISGylation/ubiquitination or promoting IκBα degradation to amplify NF-κB signaling (24, 127, 147, 151). Notably, the regulatory effects of HECT-type E3 ubiquitin ligases in macrophages are consistent across different tissue-resident macrophage subsets, with no significant functional heterogeneity between M1/M2 polarized macrophages reported to date (187).

Dendritic cells: As the only professional antigen-presenting cells initiating naive T cell activation, the functions of HECT-type E3 ubiquitin ligases in DCs are non-redundant from other cell subsets, mainly focusing on antigen presentation, type I interferon production and innate-adaptive immune crosstalk (57, 59). HERC5 is the most representative DC-specific regulatory molecule, which enhances DC antigen presentation via ISGylation modification, a function not observed in macrophages (60, 61). HERC2 and NEDD4–1 regulate type I interferon production via the TBK1-IRF3 pathway, while ITCH controls DC maturation and migration via the Notch pathway, collectively bridging innate immune sensing and adaptive immune activation (37, 90).

T lymphocytes: As the core effector cells of adaptive immunity, the functions of HECT-type E3 ubiquitin ligases in T cells are almost non-overlapping with innate immune cells, mainly focusing on T cell activation threshold, subset differentiation and immune tolerance maintenance (105, 121). ITCH is the core regulatory molecule in this cell subset, which is the only HECT-type E3 ubiquitin ligase with a non-redundant role in maintaining peripheral immune tolerance via regulating Th2/Th17 differentiation and Treg cell function (104, 105, 122). NEDD4-1, SMURF1/2 and WWP2 act as auxiliary regulators, by modulating TCR signaling and TGF-β pathway activity to fine-tune T cell-mediated adaptive inflammation (111, 123, 185). Notably, no HECT-type E3 ubiquitin ligase has been reported to exert a pro-inflammatory effect in T cells, which is a distinct characteristic from other cell subsets.

Epithelial/endothelial cells: As the physical and immune barrier of the body, HECT-type E3 ubiquitin ligases in these cells mainly regulate barrier integrity, inflammatory signal amplification and tissue fibrosis, with distinct substrate preferences from immune cells (89, 95, 148). In this cell subset, the same HECT-type E3 ubiquitin ligase most frequently exhibits opposite regulatory effects to those in immune cells. For example, HERC2 exerts an anti-inflammatory effect in macrophages but a pro-inflammatory antiviral effect in hepatocytes (37, 54); NEDD4L inhibits inflammation in immune cells but promotes inflammatory activation in renal podocytes (12, 97); HECTD3 inhibits inflammation in macrophages but promotes neuroinflammation in neurons (153, 154). This functional reversal is the most prominent feature of HECT-type E3 ubiquitin ligases in parenchymal cells (186).

The cell type-specific functions of HECT-type E3 ubiquitin ligases are critical for their clinical translation as therapeutic targets (188, 189). Systemic modulation of HECT-type E3 ubiquitin ligase activity may lead to opposite therapeutic effects in different tissues: for example, systemic inhibition of ITCH may alleviate macrophage-mediated inflammation but break T cell immune tolerance, exacerbating autoimmune diseases (103, 105, 107). Therefore, cell type-specific targeted delivery strategies based on the regulatory rules clarified in this review are the core direction for the development of HECT-type E3 ubiquitin ligase-targeted anti-inflammatory therapies (190, 191).

### 5.5 Core molecular mechanisms of HECT-type E3 ubiquitin ligases in inflammatory regulation

HECT-type E3 ubiquitin ligases are the only known family of E3 ubiquitin ligases that catalyze ubiquitin transfer via a covalent thioester intermediate, and the functional diversity of this family in inflammation regulation is fundamentally dictated by the specificity of ubiquitin chain linkage types (1, 5). The linkage type of polyubiquitin chains, defined by the lysine residue on ubiquitin that mediates chain elongation, fundamentally determines the biological outcome of ubiquitination modification (192). Among the polyubiquitination events catalyzed by HECT-type E3 ligases in inflammation, K48- and K63-linked polyubiquitin chains are the most prevalent with markedly distinct functions: K48-linked polyubiquitination is the most ubiquitous degradative ubiquitin modification in eukaryotic cells (12, 158). Once catalyzed by HECT-type E3 ligases, it is specifically recognized by the 26S proteasome to mediate proteasome-dependent degradation of substrates. In inflammatory pathways, this modification predominantly terminates or attenuates pro-inflammatory signal transduction by degrading rate-limiting kinases, transcription factors, and core inflammasome components, serving as a key negative regulatory mechanism of inflammatory responses (193). K63-linked polyubiquitination is the most representative non-degradative ubiquitin modification, which does not trigger proteasomal degradation of substrates, but acts as a molecular scaffold to mediate protein-protein interactions, signal complex assembly, and substrate subcellular localization (192). In inflammatory signaling, this modification achieves amplification and fine-tuning of pro-inflammatory signals by enhancing the kinase activity of key signaling molecules, promoting inflammasome complex assembly, and activating downstream transcription factors. In addition, HECT-type E3 ligases can also catalyze atypical non-degradative polyubiquitination including K27-, K29-, K33-linked and linear ubiquitin chains, which exert specific regulatory roles in inflammatory pathways via non-degradative mechanisms (194).

Based on the functional divergence determined by ubiquitin chain linkage types, the inflammatory regulation mediated by HECT-type E3 ligases can be clearly classified into two intrinsically distinct core modes: proteasomal degradation-dependent regulation and non-degradative signaling modulation, with fundamentally different regulatory patterns and biological functions (5, 181, 186). Degradation-dependent regulation is almost exclusively mediated by K48-linked (and a small subset of K29-linked) polyubiquitination, with the core feature of complete ablation of substrate biological functions via irreversible clearance of target proteins (14, 143, 194). In inflammation regulation, this mechanism mainly acts as a negative regulatory node: it can terminate excessive inflammatory responses by degrading pro-inflammatory signaling molecules such as IκBα, TBK1 and NLRP3, and can also promote inflammation by degrading anti-inflammatory signaling molecules, serving as a long-term, irreversible regulatory mode to maintain inflammatory signal homeostasis (90, 106). Non-degradative signaling modulation is driven by K63-, K27-, K33-linked polyubiquitination, as well as the ubiquitin-like modification ISGylation mediated by interferon-stimulated gene 15 (ISG15) (24, 44). Its core feature is that it does not alter substrate protein abundance, but reversibly modulates substrate activity, protein interaction profile and subcellular localization. In inflammation regulation, this mechanism is mainly responsible for the fine-tuning and amplification of signals, acting as a rapid and reversible regulatory mode to dynamically control the initiation and duration of inflammatory signals. Typical effects include K33-linked polyubiquitination enhancing TBK1 kinase activity (37), K63-linked polyubiquitination of RIP2 facilitating NOD2-RIP2 complex assembly (107), and ISGylation enhancing NLRP3 stability to promote inflammasome activation (24).

In addition to ubiquitin chain-dependent functional divergence, adaptor proteins are core regulators that dictate the substrate specificity and catalytic activity of HECT-type E3 ligases, as well as indispensable components for their inflammatory regulatory functions, with two major functional dimensions: substrate targeting and activity regulation (73, 186). In terms of substrate targeting, HECT-type E3 ligases can recognize substrates via their intrinsic characteristic domains (e.g., WW domains of the NEDD4 subfamily, RLD domains of the HERC subfamily) (13, 18, 20). For substrates lacking canonical binding motifs, adaptor proteins act as a “molecular bridge” that simultaneously binds to the HECT-type E3 ligase and the substrate protein, shortening the spatial distance to achieve site-specific ubiquitination of the substrate. This mechanism endows HECT-type E3 ligases with cell type-specific and microenvironment-dependent substrate selectivity during inflammatory responses, with typical examples including TMEPAI recruiting NEDD4–1 to mediate IκBα ubiquitination (96), and ribosomal protein Rps3 mediating the interaction between HECTD1 and IκBα and subsequent ubiquitination modification (147). In terms of catalytic activity regulation, most HECT-type E3 ligases maintain an autoinhibited conformation via intramolecular interactions in the resting state. Adaptor proteins can bind to their regulatory domains to relieve autoinhibition and significantly enhance catalytic activity, serving as a core molecular switch that controls the initiation and termination of HECT-type E3 ligase-mediated inflammatory regulation (78, 195). For instance, Ndfip family proteins can relieve the autoinhibition of NEDD4–1 and ITCH, and TMEPAI can simultaneously achieve substrate recruitment and catalytic activity enhancement of NEDD4-1. Collectively, the three core mechanisms described above constitute a unified framework for the functional diversity of HECT-type E3 ligases in inflammation regulation, and provide a systematic mechanistic reference for subsequent functional characterization of individual family members (5, 186, 196).

## 6 Potential and challenges of HECT-type E3 ubiquitin ligases as therapeutic targets for inflammation

HECT-type E3 ubiquitin ligases are advancing rapidly in therapeutic development and represent important therapeutic targets due to their regulatory roles in disease pathogenesis (197). In recent years, strategies to modulate HECT-type E3 ubiquitin ligase function have expanded to include small-molecule inhibitors and targeted protein degradation technologies such as Proteolysis-Targeting Chimeras (PROTACs). Small-molecule inhibitors predominantly target the HECT domain, particularly its catalytic active sites (188, 189, 198). Studies have shown that natural products, including indole-3-methanol and its derivatives, can specifically inhibit the catalytic activity of WWP1 and WWP2 and block their ubiquitin ligase function. These findings highlight their potential antitumor efficacy (199). In addition, synthetic small molecules can selectively inhibit the ubiquitination activity of HUWE1, thereby suppressing tumor cell proliferation. PROTAC technology offers a novel strategy to modulate target proteins, including membrane-associated proteins, by recruiting endogenous HECT E3-type ubiquitin ligases to mediate the degradation of specific substrates (200).

Preclinical studies have demonstrated that HECT E3 ubiquitin ligase inhibitors hold therapeutic potential across various disease models, most notably in hepatocellular carcinoma and viral infections (201–203). However, current research on the translational application of HECT-type E3 ubiquitin ligases as inflammatory therapeutic targets has four key limitations: first, the lack of subtype-specific inhibitors, with most existing inhibitors being broad-spectrum inhibitors of the HECT domain, unable to distinguish between different members of the HECT family, leading to serious off-target effects and potential systemic immune imbalance risks; second, the bidirectional inflammatory regulatory function of most HECT-type E3 ubiquitin ligases in different cell types has not been fully considered, and non-cell-specific targeted regulation may lead to opposite therapeutic effects in different tissues; third, almost all studies are limited to cell and mouse preclinical models, with no clinical trial data in inflammatory diseases, and the pharmacokinetic properties, *in vivo* delivery efficiency, and safety of the inhibitors have not been verified in humans; fourth, the research on the biomarker value of HECT-type E3 ubiquitin ligases in inflammatory diseases is almost blank, with no large-scale clinical cohort verification of their correlation with disease diagnosis, prognosis, and treatment response. Although inhibitors targeting HECT-type E3 ubiquitin ligases exhibit high selectivity and specificity *in vitro* experiments, their clinical translation still faces substantial challenges, including the development of selective inhibitors, suboptimal pharmacokinetic properties, and safety concerns. Therefore, future investigations should focus on integrating structural biology with high-throughput screening to develop more potent and subtype-specific modulators, and carry out cell type-specific targeted delivery design based on the cell context-dependent function of HECT-type E3 ubiquitin ligases. Concurrently, multidisciplinary collaboration will further advance our understanding of the molecular mechanisms underlying HECT-type E3 ubiquitin ligase function and promote their application in the treatment of inflammatory diseases, with the ultimate goal of yielding more effective therapeutic strategies (190, 191, 204).

## 7 Conclusion

HECT-type E3 ubiquitin ligases are key regulatory factors in inflammatory and immune signaling networks, and their unique domain architectures enable the selective targeting of multiple substrates. They exert pivotal regulatory roles in modulating the NF-κB signaling, inflammasome activation, and pro-inflammatory cytokine production, thereby highlighting the functional versatility of the HECT-type E3 ubiquitin ligase superfamily (5). This review systematically summarizes the research progress of the HERC subfamily, NEDD4 subfamily, and other HECT members in inflammatory regulation, and further conducts a critical analysis of the current research status in this field. First, there are widespread conflicting research conclusions in existing studies, with most HECT family members showing bidirectional inflammatory regulatory effects, which are mainly derived from the neglect of their cell type context-dependent functions; second, current research has significant limitations, including insufficient verification in physiological models, lack of cell type-specific *in vivo* studies, scarce research on non-classical ubiquitin modifications, and huge gaps in clinical translational research; third, the inflammatory regulatory function of specific HECT-type E3 ubiquitin ligases shows strict cell type context dependence, which is determined by cell-specific substrate expression, ubiquitin chain type preference, subcellular localization, and upstream regulatory signals. Although recent studies have begun to unravel the molecular mechanisms of HECT-type E3 ubiquitin ligases, the heterogeneity across studies underscores the need to better define their spatiotemporal dynamics, cell-type specificity, and context-dependent substrate selection during inflammatory responses. A deeper mechanistic understanding will help establish a comprehensive and accurate model of inflammatory regulation (186). Moving forward, mechanistic dissection of HECT-type E3 ubiquitin ligases in inflammatory diseases especially the clarification of their cell type-specific regulatory mechanisms, resolution of conflicting research conclusions, and breakthrough of clinical translational bottlenecks, is expected to provide critical insights for precision medicine and facilitate the identification of disease-specific biomarkers. These advances will promote their translational application in the diagnosis and treatment of inflammatory disorders, expand the investigative scope of inflammatory biology, and open new avenues for the development of novel anti-inflammatory therapeutic strategies (196, 205).
